# Incidence and Treatment of Arginine Vasopressin Deficiency (Central Diabetes Insipidus) in the Setting of Brain Death and Associations with Renal Function and Hemodynamics in Organ Donors

**DOI:** 10.3390/jcm13237073

**Published:** 2024-11-22

**Authors:** Marleen Weiß, Fabian Rücker, Volker Thieme, Karsten Hochmuth, Dominik Michalski, Björn Nashan, Hans-Michael Tautenhahn, Robert Werdehausen, Svitlana Ziganshyna

**Affiliations:** 1 Department of Anesthesiology and Intensive Care Medicine, Medical Faculty, Leipzig University, University of Leipzig Medical Center, 04103 Leipzig, Germany; marleen.weiss@med.ovgu.de (M.W.);; 2 Department of Anesthesiology and Intensive Care Medicine, Medical Faculty, University of Magdeburg, 39120 Magdeburg, Germany; robert.werdehausen@med.ovgu.de; 3 German Organ Procurement Organization (DSO), Region East, 04157 Leipzig, Germany; 4 Department of Neurology, University of Leipzig Medical Center, 04103 Leipzig, Germany; dominik.michalski@medizin.uni-leipzig.de; 5 Department of Organ Transplantation Center, The First Affiliated Hospital of University of Science and Technology of China, Hefei 230001, China; bjoern.nashan@gmail.com; 6 Department of Visceral, Transplantation, Thoracic and Vascular Surgery, University of Leipzig Medical Center, 04103 Leipzig, Germany; hans-michael.tautenhahn@medizin.uni-leipzig.de; 7 Organ Donation Coordinator Unit, Medical Faculty, Leipzig University, University of Leipzig Medical Center, 04103 Leipzig, Germany

**Keywords:** organ protection, central diabetes insipidus (CDI), arginine vasopressin deficiency (AVP-D), desmopressin, acute kidney injury, transplantation, donors, brain death

## Abstract

**Background/Objectives**: Arginine vasopressin deficiency (AVP-D) is a common condition in the setting of brain death. The aim of this study was to analyze the frequency of AVP-D in organ donors, its treatment, as well as the impact of AVP-D on hemodynamics and renal function. **Methods**: This single-center, retrospective study included 63 organ donors treated between 2017 and 2022. We used standard criteria to examine the incidence of AVP-D and the KDIGO criteria to determine the rate of acute kidney injury (AKI). **Results**: AVP-D occurred in 79% of the examined organ donors, of which 94% received desmopressin. Overall, 30% of organ donors developed AKI. AKI was present in 77% of donors who did not meet AVP-D criteria and in only 18% of donors with AVP-D (*p* < 0.001). Mean arterial blood pressure did not differ between organ donors with and without AVP-D or with and without desmopressin therapy. In organ donors with AVP-D, norepinephrine requirement in the period 24 h prior to AVP-D diagnosis was lower than 24 h afterwards (*p* = 0.03). AVP-D diagnosis was associated with a higher rate of kidney transplantation compared to cases without AVP-D diagnosis (88% vs. 54%, *p* = 0.01). **Conclusions**: AVP-D is common among brain death organ donors and may remain undiagnosed in cases with previous kidney injury. These observations highlight the importance of recognizing AVP-D and administering appropriate therapy in potential organ donors to prevent AKI.

## 1. Introduction

According to Eurotransplant, at the end of year 2023, there were 13,498 patients on the active waiting list, of whom 10,404 were waiting for a kidney. Out of 5639 organs transplanted from January to November 2023 from deceased donors, 2862 were kidneys [1]. These data clearly indicate a critical shortage of organs.

There is limited knowledge on the optimal organ-protective therapy for patients with brain death being considered for organ donation. Most recommendations are based on pathophysiological considerations and expert opinions. Evidence-based recommendations are thus needed for more comprehensive organ-protective therapy, which might increase the number of organs transplanted per donor and improve organ function.

Brain death results in pathophysiological changes in the donor. These affect the hormonal balance, hemodynamics, body temperature, and pulmonary function [2]. One of these changes is the occurrence of arginine vasopressin deficiency (AVP-D), previously known as central diabetes insipidus (CDI). The change in terminology aimed to clearly depict the pathophysiological process leading to AVP-D. The latter is a result of impaired production of ADH, a consequence of disordered hypothalamus and pituitary gland function [3]. ADH is produced in the supraoptic and paraventricular nuclei in the hypothalamus and secreted out of the posterior pituitary gland. It acts as a vasoconstrictor via V1 receptors on vascular muscle cells, leading to an increase in blood pressure. At the V2 receptors, ADH stimulates water reabsorption via the incorporation of aquaporins in the collecting duct. In the absence of ADH, severe diuresis, followed by hypovolemia and hypotension, occur [4], which leads to inadequate organ perfusion and pre-renal kidney failure [3]. Acute kidney failure is one reason for the loss of potential donor organs before retrieval [4].

Since the kidney is the most sought-after organ, the early detection of AVP-D and treatment with desmopressin are crucial. Fluid therapy with crystalloid solutions is necessary to treat hypovolemia [5], enabling sufficient renal perfusion through euvolemia and normotension. For some donors, however, catecholamines, especially norepinephrine, are required to achieve an adequate perfusion pressure. Individual goals include a mean arterial blood pressure (mABP) of 60–70 mmHg and a urine output volume of 1–3 mL/kg body weight (BW)/h [6]. Therefore, continuous monitoring of hemodynamic parameters is highly recommended.

The available data on the incidence of AVP-D varies widely: in a review by Hahnenkamp et al., an AVP-D frequency of 65–90% of all donors was reported [7]. A publication by Salim et al. noted a frequency of 46.6% of all donors [8], while a study by Gramm et al. found that approximately 78% of donors developed AVP-D [9]. Further, Yener et al. investigated the occurrence of AVP-D in children with brain death. In this study, 89% of those examined developed AVP-D [10].

The highly varying incidence of AVP-D along with brain death supports the need for further research. On the individual level, it is important to recognize and treat AVP-D early in order to prevent consequences such as prerenal kidney failure. With desmopressin, an exogenously administered analog of ADH, there is a simple and effective treatment for AVP-D [11].

The aim of this retrospective study was to determine the frequency of AVP-D in organ donors and to analyze how often AVP-D was treated and when therapy was initiated with relation to the time of its diagnosis. In addition, the impact of AVP-D on hemodynamics, catecholamine requirements, and renal function of organ donors was evaluated. Finally, we aimed to determine the transferability of our findings with regard to the rate of organ allocation and transplantation into clinical practice.

## 2. Materials and Methods

After receiving approval from the responsible local ethics committee of the Medical Faculty of the University of Leipzig (reference number 397/22-ek, 8 November 2022), data from 63 organ donors with confirmed brain death between January 2017 and April 2022 were examined in a monocentric (University of Leipzig Medical Center) retrospective study. In this analysis, we reviewed the electronic medical records beginning from at least 7 days prior to brain death.

The diagnosis of AVP-D in organ donors was based on the following criteria:

(1) A serum sodium level higher than 145 mmol/L.
(2) Diuresis > 3 mL/kg BW/h in donors aged 2 years and older [5] or >4 mL/kg BW/h in donors younger than 2 years [10].
(3) Exclusion of other causes of increased diuresis and hypernatremia, including hyperglycemia in the context of osmotic diuresis (assessed by measuring blood glucose), elevated calcium or potassium levels, and the administration of hyperosmolar (e.g., mannitol) or hypertonic fluids.
(4) When available, urine osmolality < 300 mOsmol/L, blood osmolality > 300 mOsmol/L [3], and specific urine weight < 1005 [5].

Only a limited number of urine and blood osmolality measurements were available in our cohort. We calculated the diuresis limits based on the organ donor’s body weight. Once diuresis increased over two consecutive time periods, the criterion of polyuria was met. Other causes of increased diuresis and hypernatremia, such as hyperglycemia in the context of osmotic diuresis, measured blood glucose, calcium, potassium, and administration of hyperosmolar (e.g., mannitol) or hypertonic fluids, could be ruled out.

Furthermore, the number of patients treated with desmopressin was recorded, and the duration between fulfilling the AVP-D criteria and commencement of therapy was analyzed. There was no established standard protocol for the treatment of AVP-D in place at the contributing intensive care units. Desmopressin dose ranged from 2 to 5 µg applied intravenously or nasally per application. Those donors were divided into three groups: those who prematurely received therapy at the discretion of the managing physicians, i.e., before fulfilling all AVP-D criteria, those within six hours of meeting AVP-D criteria, and those with a delay of more than six hours from the time of meeting AVP-D criteria.

To determine how many of the donors developed acute kidney injury (AKI), we used the serum creatinine values based on the Kidney Disease: Improving Global Outcomes (KDIGO) criteria [12]. We also examined the number of kidneys allocated and transplanted per donor. Subsequently, we compared the number of donors with at least one transplanted kidney in different groups.

We recorded norepinephrine doses and mABP before and after the diagnosis of AVP-D, as well as before and after desmopressin administration. This was carried out in order to assess the impact of AVP-D on the hemodynamics of organ donors. Additionally, the norepinephrine requirement in the three subgroups relating to the timing of therapy was analyzed. For this purpose, we determined the norepinephrine dose and the mABP averaged over 24 h before and after the onset of AVP-D. The same procedure was used to determine the average norepinephrine dose 24 h before and after the first desmopressin administration. Finally, we compared the medians of norepinephrine doses in donors with and without AVP-D as well as those with and without desmopressin treatment. In the statistical analysis, we determined the median values of the data in each of the different groups and defined the 25th and 75th percentiles as measures of dispersion. These are noted in parentheses after the median.

Statistical analysis was performed with the software Prism (version 10.3, Graphpad, La Jolla, CA, USA). For testing the statistical significance between groups, the Mann–Whitney test, Wilcoxon test, or Fisher’s exact test (all two-tailed) were applied as indicated, depending on the type of comparison. Thereby, a *p*-value < 0.05 was considered indicative of statistically significant differences.

## 3. Results

### 3.1. Demographic Data

Our organ donors’ cohort consisted of 33 females and 30 males (Table 1), of which four were children under the age of 18 years. The cohort’s median age was 59 years (range: 3–90), while the median weight was 80 kg (range: 15–130). The etiology of brain death was intracranial hemorrhage in 54%, ischemic–hypoxic brain injury in 19%, traumatic brain injury in 12.7%, stroke in 11.1%, or other causes such as intracranial infections and tumors in 3.2% of all included cases.

### 3.2. Incidence and Treatment of AVP-D

**Figure 1:**
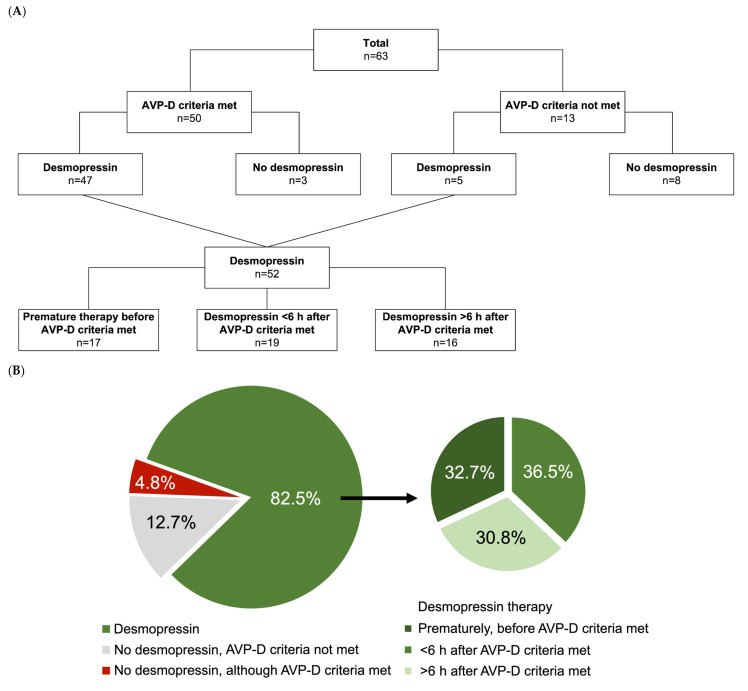
Occurrence of arginine vasopressin deficiency (AVP-D) and subsequent initiation rates for desmopressin therapy in brain dead organ donors. (**A**) depicts the proportions of donors with fulfilled AVP-D criteria and the resulting subgroups of donors with and without desmopressin therapy. (**B**) is a graphical representation of the proportion of donors who received desmopressin therapy in the left pie chart, while on the right the percentage of donors whose desmopressin therapy was initiated prematurely, within 6 h, or later than 6 h after meeting AVP-D criteria is illustrated.

The predefined criteria for AVP-D were met in 50 out of the total 63 (79.4%) of the examined organ donors (Figure 1A). Among those meeting the AVP-D criteria, 47 out of 50 (94.0%) received desmopressin therapy, while five patients not fulfilling the AVP-D criteria also received desmopressin (Figure 1B). Seventeen of those 52 organ donors (32.7%) who were administered desmopressin were treated prematurely, even though not all AVP-D criteria were met at the point of therapy. In 19 out of 52 AVP-D-treated cases (36.5%), therapy with desmopressin was initiated within 6 h of AVP-D diagnosis, while in another 16 cases (30.8%), therapy commenced after 6 h. Three patients (4.8%) did not receive treatment despite meeting all AVP-D criteria. The main reasons for the initiation of therapy with desmopressin were hypernatremia and polyuria.

### 3.3. Impact on Renal Function

**Figure 2:**
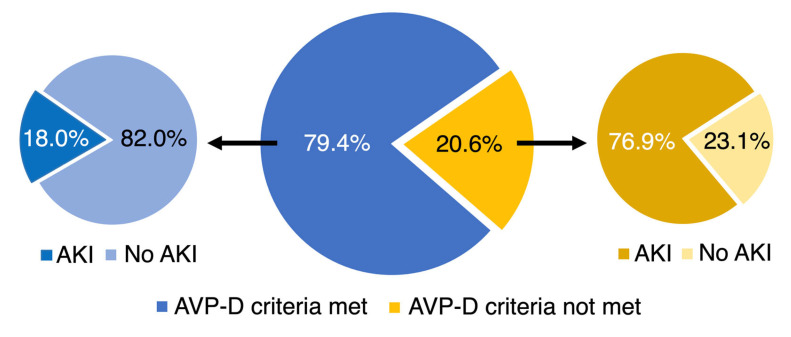
Prevalence of acute kidney injury (AKI) with respect to arginine vasopressin deficiency (AVP-D). The incidence of AVP-D in our cohort is shown in the main pie chart. The prevalence of acute kidney injury as a proportion of donors with AVP-D is depicted in the left diagram versus those organ donors without AVP-D diagnosis on the right.

Regarding diuresis, a marked decline in urine output in association with desmopressin therapy was observed. Prior to desmopressin administration, the median urine output was 5.3 (3.1–7.1) mL/kg BW/h, which was reduced to 1.6 (1.0–3.2) mL/kg BW/h afterwards (*p* < 0.01). Overall, 19 out of 63 (30.2%) organ donors developed AKI according to KDIGO criteria (Figure 2). Of the 13 organ donors who did not fulfill the criteria for AVP-D, ten (76.9%) donors developed acute kidney injury (Table 1). Furthermore, nine out of 50 (18.0%) organ donors who met AVP-D criteria developed AKI, which occurred in four (44.4%) cases after the AVP-D criteria were met. Among the donors who did not meet AVP-D criteria, 69.2% had an early AKI (9 out of 13).

### 3.4. Impact on Hemodynamics

Mean arterial blood pressure did not differ significantly between organ donors with and without AVP-D (83 (78–90) vs. 78 (75–86) mmHg; *p* = 0.08) or with and without desmopressin therapy (82 (77–89) vs. 81 (75–98) mmHg; *p* = 0.83).

**Figure 3:**
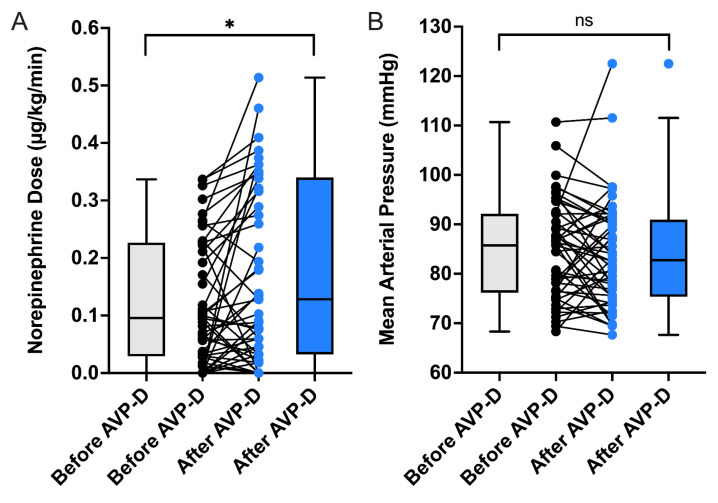
Effects of arginine vasopressin deficiency (AVP-D) on the norepinephrine requirement in relation to mean arterial blood pressure. (**A**) depicts the significantly increased norepinephrine dose in the period following AVP-D diagnosis compared to the 24-h period prior to diagnosis. * *p* < 0.05; Wilcoxon test. (**B**) shows the effect of AVP-D on the mean arterial blood pressure of all donors with AVP-D.

The median dose of continuous norepinephrine application over the entire observation period was 0.15 (0.09–0.24) µg/(kg·min) in organ donors with AVP-D. In donors without AVP-D, the median dose over the entire data collection period was 0.19 (0.13–0.41) µg/(kg·min); (*p* = 0.17). In the 24-h period prior to AVP-D diagnosis, the median dose of norepinephrine required was 0.10 (0.03–0.23) µg/(kg·min) and increased to a median of 0.13 (0.03–0.34) µg/(kg·min) 24 h after AVP-D diagnosis (*p* = 0.03; Figure 3A). However, the mean arterial blood pressure did not significantly fall in this context (86 vs. 83 mmHg; *p* = 0.3; Figure 3B).

Donors who received desmopressin therapy had a median norepinephrine requirement of 0.16 (0.10–0.25) µg/(kg·min), while those without treatment had a median norepinephrine requirement of 0.19 (0.08–0.37) µg/(kg·min); (*p* = 0.62). In donors with desmopressin therapy, the norepinephrine dose recorded over a 24-h period following the start of therapy remained constant at a median of 0.09 (0.04–0.25) µg/(kg·min) compared to the 24-h period prior to the initiation of treatment (0.09 (0.01–0.23) µg/(kg·min); *p* = 0.57). Interestingly, when we compared subgroups based on the timing when desmopressin therapy was initiated (prematurely, within 6 h after fulfilling AVP-D criteria, or later), the norepinephrine dose did not significantly differ within a 24-h period before and after therapy commencement (*p* = 0.89; *p* = 0.28; *p* = 0.95, respectively).

### 3.5. Impact on Allocation and Transplantation of Organs

In total, 107 of 126 kidneys (85%) were entered into the allocation process. The remaining could not be entered because of acute kidney injury (6%) or chronic kidney disease (9%). Further 10 kidneys (8%) could not be transplanted after entering the allocation because of atherosclerotic graft vessels (n = 4), malignancy (n = 4), or unsuccessful allocation (n = 2).

An AVP-D diagnosis was associated with organ allocation in 46 out of 50 cases (92%) and with the transplantation of at least one kidney in 44 out of 50 cases (88%) (both *p* = 0.01) (Table 1). Without an AVP-D diagnosis, allocation occurred in only 8 out of 13 cases (62%), and transplantation of at least one kidney occurred in 7 out of 13 cases (54%) (both *p* = 0.01).

In donors with desmopressin therapy, an allocation of at least one kidney was achieved in 47 out of 52 cases (90%) compared to 7 out of 11 cases (64%) without desmopressin therapy (*p* = 0.04).

At least one kidney could be transplanted in 45 of 52 donors (87%), who received desmopressin therapy, while only 5 of 11 cases (45%) were transplanted without desmopressin therapy (*p* = 0.01). Comparing the different subgroups by time of therapy initiation, no significant differences were found in the proportion of kidneys allocated or transplanted.

## 4. Discussion

A variety of pathophysiological changes occur in organ donors in association with brain death; for instance, a disturbed production of ADH often leads to AVP-D. In the study by Gramm et al., 78% of the examined donors developed AVP-D [9]. In our study, almost 80% of the organ donors examined also fulfilled the defined AVP-D criteria. This included hypernatremia (>145 mmol/L) and polyuria with a urine output greater than 3 mL/kg BW/h or 4 mL/kg BW/h in children under 2 years of age. The criteria for the diagnosis of AVP-D were selected based on the referenced literature and the available data and clinical criteria, as used at the involved intensive care units.

Given the pathophysiological processes accompanying brain death, one would expect that all organ donors will develop AVP-D. However, a relevant proportion of 17 donors (27%) in our cohort were treated prematurely, i.e., before all AVP-D criteria were fulfilled. This premature treatment most probably prevented a full clinical manifestation of AVP-D in five of these donors, likely leading to an underestimate of the incidence of AVP-D.

Additionally, preexisting kidney disease or early occurrence of AKI may have complicated AVP-D recognition. Examining the organ donors’ renal function, we observed that approximately one-third of all donors met the KDIGO criteria for an AKI. Notably, there was a significantly lower rate of AKI in donors with an AVP-D diagnosis (18.0%) compared to those who did not meet the AVP-D criteria (76.9%). Nine out of 10 donors without fulfilled AVP-D criteria developed early AKI, which we defined as occurring within 24 h of admission. The one donor without early AKI already showed elevated baseline serum creatinine and potassium levels at the time of admission. However, there was an insufficient increase in the serum creatinine level during the course of hospitalization to meet the KDIGO criteria. In the three donors who did not have AKI or AVP-D, organ recovery was performed early, within 48 h of admission. One of these donors exhibited polyuria and hypernatremia shortly before organ recovery. Nonetheless, organ recovery occurred before the second polyuric measurement, necessary to meet the AVP-D criteria, was met.

Considering the hemodynamics of donors with AVP-D, one could expect that the mABP in the 24-h period after AVP-D diagnosis would be lower than in the preceding 24 h, due to polyuria. Nevertheless, there was no significant difference in our study. This can be attributed to the administration of higher norepinephrine doses in the 24-h period after AVP-D diagnosis. As a result, the mABP was kept constant and adequate organ perfusion was achieved, despite the hypovolemia and hypotension associated with AVP-D. The donors received mainly norepinephrine and, rarely, epinephrine. However, we only considered the continuous administration of norepinephrine, since epinephrine was only administered in the form of a single bolus application in the context of critical events to a small proportion of donors.

Desmopressin therapy was performed in 94% of patients with fulfilled AVP-D criteria. The majority of donors who received desmopressin were treated either prematurely (32.7%), i.e., before meeting all AVP-D criteria, or early, i.e., within 6 h (36.5%) of fulfilling AVP-D criteria. In the remaining donors (30.8%), desmopressin therapy was commenced later than 6 h after receiving the AVP-D diagnosis. Only 6% of donors with fulfilled AVP-D criteria were not treated. The mABP did not differ between donors with and without AVP-D or those with and without desmopressin therapy. Probably because of the high proportion of desmopressin therapy (94%) in donors with AVP-D, the polyuric period in the context of AVP-D could have been shortened or even prevented, leading to a nephroprotective effect. Consequently, the rate of AKI in the group of donors meeting AVP-D criteria was almost 60% lower compared to those without AVP-D. Adequate desmopressin therapy also attenuated the effects of AVP-D on the donors’ hemodynamics. In our study, desmopressin therapy was associated with a significantly higher rate of allocated and transplanted kidneys per donor, and these results are supported by the findings of Callahan et al. [13]. They were able to show that desmopressin reduced volume requirements, thus improving the number of transplanted organs and the recovery of lung function. Guesde et al. investigated the use of desmopressin therapy in donors with brain death in 1998 and its impact on renal function and outcomes after transplantation in organ recipients [14]. With regard to creatinine concentration and long-term transplant survival, no significant difference was found between the group of donors that received desmopressin therapy and those without treatment. However, in 2004, Niijboer et al. demonstrated that one to three years following transplantation, kidney organ recipients had lower serum creatinine levels and a decreased risk of rejection when kidney donors received desmopressin [15]. Benck et al. also showed that desmopressin therapy improves the survival of kidney transplants [11].

In our study, we were able to show a high incidence (79.4%) of AVP-D in the setting of brain death, while its absence was probably due to other confounding factors such as AKI. Some donors did not meet the AVP-D criteria, as the criterion of polyuria necessary to diagnose AVP-D could not be fulfilled, presumably due to reduced diuresis in the context of AKI. The latter as well as prompt organ recovery can complicate the diagnosis of AVP-D in the context of brain death since the criterion of polyuria may not always be met. In support of our findings, another very recent publication by Varelas PN et al. also reported a relatively low frequency of AVP-D in adult organ donors and highlighted a reduced glomerular function as the cause for less frequent clinical manifestations of AVP-D in organ donors [16].

Furthermore, we were able to demonstrate that early AKI, occurring within 24 h after admission, especially in the group of donors who did not meet the AVP-D criteria, leads to a low rate of allocated and transplanted kidneys.

Despite having a modest number of participants, the main strength of this study lies in the data quality we were able to collect over a 5-year study period. The spectrum and proportions of brain death etiologies in our cohort were representative of the overall collective of brain-dead organ donors based on Organ Procurement and Transplantation Network (OPTN) data as of 10 April 2024 (http://optn.transplant.hrsa.gov). No donors needed to be excluded due to missing data regarding AVP-D diagnosis, treatment with desmopressin, or norepinephrine dosages. Nonetheless, this study was limited by its retrospective, monocentric nature.

The high rate of premature or early desmopressin therapy in organ donors indicates that there is already effective screening and management of donors at the participating intensive care units. Still, efforts should continue to focus on early detection and treatment of AVP-D. In addition to the clinical criteria currently used, serum copeptin measurement has been suggested as a promising diagnostic tool for AVP-D, particularly in cases with ambiguous clinical criteria [17]. Methods such as hypertonic saline or arginine-stimulated copeptin tests demonstrate high diagnostic accuracy but are challenging to apply in critically ill ICU patients with severe brain injury. These tests are time-intensive and may risk complications such as hypernatremia, potentially interfering with brain death diagnostics. Although not available in our cohort, further methodological refinements could facilitate their future use in brain-dead organ donors.

Our study indicates that the use of desmopressin could result in a lower rate of AKI and a higher number of transplanted kidneys. Optimal donor management in the intensive care unit is important to minimize donor or organ loss [18]. Consequently, guidelines for the management of potential organ donors should be developed and acknowledge the importance of close organ function monitoring and protective interventions in potential organ donors [5,19].

Future research should adopt a prospective, observational study design with predefined monitoring and treatment protocols to better understand the benefits of early detection and treatment of AVP-D. However, because of the relatively low number of brain death cases, a multicentric approach appears necessary. It seems also worth considering a study with active interventions to explore how the preemptive administration of desmopressin affects the number of available kidney donors and the long-term success of transplantation.

In conclusion, AVP-D is a common condition associated with brain death that, due to reduced diuresis resulting from renal dysfunction, may remain undetected. This highlights the importance of vigilant monitoring in brain-dead donors to ensure early detection and management of AVP-D.

## Figures and Tables

**Table 1:** Demographic data from an organ donor cohort with and without met criteria for arginine vasopressin deficiency AVP-D. The median duration between the time of admission and the determination of brain death was three days, with a minimum and maximum duration of 1 and 15 days, respectively.

Organ Donor Characteristics	AVP-D	No AVP-D	* p * -Value
Overall, *n* (%)	50 (79)	13 (21)	-
Female sex, *n* (%)	27 (54)	6 (46)	0.76
Median age (y)	57	66	0.30
Minimum age (y)	3	7	
Maximum age (y)	90	81	
Median weight (kg)	75	80	0.93
Minimum weight (kg)	15	20	
Maximum weight (kg)	130	86	
Etiology			
Intracranial hemorrhage, *n* (%)	29 (58)	5 (38)	0.23
Ischemic hypoxic brain injury, *n* (%)	7 (14)	5 (38)	0.11
Traumatic brain injury, *n* (%)	6 (12)	2 (15)	0.66
Stroke, *n* (%)	6 (12)	1 (8)	>0.99
Other, *n* (%)	2 (4)	0 (0)	>0.99
Acute kidney injury (AKI), *n* (%)	9 (18)	10 (77)	<0.001
Kidney(s), allocated			0.01
One kidney, *n* (%)	0 (0)	0 (0)	
Both kidneys, *n* (%)	46 (92)	8 (62)	
Kidney(s), transplanted			0.01
One kidney, *n* (%)	3 (6)	0 (0)	
Both kidneys, *n* (%)	41 (82)	7 (54)	

## Data Availability

The data presented in this study are available upon request from the corresponding author due to legal and ethical restrictions related to deceased organ donors and organ allocation.
